# Targeted Gene Sequencing in Children with Crohn’s Disease and Their Parents: Implications for Missing Heritability

**DOI:** 10.1534/g3.118.200404

**Published:** 2018-08-30

**Authors:** Jiun-Sheng Chen, Fulan Hu, Subra Kugathasan, Lynn B. Jorde, David Nix, Ann Rutherford, Lee Denson, W. Scott Watkins, Sampath Prahalad, Chad Huff, Stephen L. Guthery

**Affiliations:** * Department of Epidemiology, The University of Texas MD Anderson Cancer Center, Houston, TX; † Biostatistics, Bioinformatics and Systems Biology Program, The University of Texas Graduate School of Biomedical Sciences at Houston and MD Anderson Cancer Center, Houston, TX; ‡ Department of Epidemiology, Public Health College, Harbin Medical University, Harbin, Heilongjiang 150081, China; § Department of Pediatrics, Emory University School of Medicine, Atlanta, GA; ** Department of Human Genetics, University of Utah School of Medicine, Salt Lake City, UT; †† Department of Population Sciences, Huntsman Cancer Institute, University of Utah School of Medicine, Salt Lake City, UT; ‡‡ Department of Pediatrics, University of Utah School of Medicine, Salt Lake City, UT; §§ Department of Pediatrics, Cincinnati Children’s Hospital Medical Center, Cincinnati, OH

**Keywords:** Crohn’s disease, *NOD2*, rare variant, *de novo* mutation, case-control study

## Abstract

Crohn’s disease is a complex genetic trait characterized by chronic relapsing intestinal inflammation. Genome wide association studies (GWAS) have identified more than 170 loci associated with the disease, accounting for ∼14% of the disease variance. We hypothesized that rare genetic variation in GWAS positional candidates also contribute to disease pathogenesis. We performed targeted, massively-parallel sequencing of 101 genes in 205 children with Crohn’s disease, including 179 parent-child trios and 200 controls, both of European ancestry. We used the gene burden test implemented in VAAST and estimated effect sizes using logistic regression and meta-analyses. We identified three genes with nominally significant p-values: *NOD2*, *RTKN2*, and *MGAT3*. Only *NOD2* was significant after correcting for multiple comparisons. We identified eight novel rare variants in *NOD2* that are likely disease-associated. Incorporation of rare variation and compound heterozygosity nominally increased the proportion of variance explained from 0.074 to 0.089. We estimated the population attributable risk and total heritability of variation in *NOD2* to be 32.9% and 3.4%, respectively, with 3.7% and 0.25% accounted for by rare putatively functional variants. Sequencing probands (as opposed to genotyping) to identify rare variants and incorporating phase by sequencing parents can recover a portion of the missing heritability of Crohn’s disease.

Crohn’s disease is an idiopathic inflammatory bowel disease (IBD) characterized by chronic and relapsing inflammation of the gastrointestinal tract. Crohn’s disease is believed to be caused by an abnormal immune response to intestinal microbiota in genetically susceptible individuals (Sartor 2006; Sartor 2008; Kostic *et al.* 2014; Sartor and Wu 2017). Twin concordance and familial aggregation studies overwhelmingly support a significant genetic contribution to Crohn’s disease (Tysk *et al.* 1988; Monsén *et al.* 1991; Orholm *et al.* 1991; Meucci *et al.* 1992; Thompson *et al.* 1996; Russel *et al.* 1997; Orholm *et al.* 2000; Halfvarson *et al.* 2003; Reynisdottir *et al.* 2004; Guthery *et al.* 2011), and genome wide association studies (GWAS) have identified susceptibility loci in affected individuals of European (Duerr *et al.* 2006; Hampe *et al.* 2007; Parkes *et al.* 2007; Rioux *et al.* 2007; Wellcome Trust Case Control Consortium 2007; Barrett *et al.* 2008; Kugathasan *et al.* 2008; Imielinski *et al.* 2009; Franke *et al.* 2010; Liu *et al.* 2011; Jostins *et al.* 2012), Asian (Yamazaki *et al.* 2005; Umeno *et al.* 2011), and African (Brant *et al.* 2017) ancestry.

Pan-ancestry GWAS have thus far identified more than 170 loci associated with Crohn’s disease (Liu *et al.* 2015). These loci are enriched for genes involved in important pathways of Crohn’s disease, including innate immunity, autophagy, and the IL-23-T_H_17 pathway(Ramjeet *et al.* 2010; Corridoni *et al.* 2014). For most of the >170 common variant associations implicated in Crohn’s disease, the causal variants responsible for disease association are unknown. Moreover, currently identified variants account for only ∼14% of the variance explained for Crohn’s disease (Barrett *et al.* 2008; Franke *et al.* 2010; Jostins *et al.* 2012).

Rare variants are potentially more deleterious than common variation due to the effects of natural selection (Eyre-Walker and Keightley 2007). Although they are individually rare, they may in aggregate contribute to Crohn’s disease and other complex genetic diseases (Eichler *et al.* 2010; Veltman and Brunner 2012). Indeed, multiple exome sequencing studies have identified rare and highly penetrant mutations in multiple genes that cause chronic intestinal inflammation (Glocker *et al.* 2009; Rivas *et al.* 2011; Worthey *et al.* 2011. ; Alangari *et al.* 2012; Christodoulou *et al.* 2013; Dinwiddie *et al.* 2013; Avitzur *et al.* 2014; Dhillon *et al.* 2014; Hu *et al.* 2014; Okou *et al.* 2014; Kelsen *et al.* 2015; Hong *et al.* 2016). Moreover, the contribution of *de novo* variation to Crohn’s disease susceptibility is currently unknown.

To further explore the contribution of rare variants and *de novo* mutations to Crohn’s disease susceptibility, we conducted targeted sequencing of 101 genes identified by GWAS in 205 childhood onset patient-offspring trios and 200 unaffected controls. To test for rare variant association in each gene, we conducted gene-based burden tests using VAAST and *de novo* mutation association tests using VARPRISM(Kong *et al.* 2012; Hu *et al.* 2016). Both tests incorporate functional variant prediction information to improve statistical power. Collectively, this study not only reaffirms the role of common variation in *NOD2* as the primary genetic risk for Crohn’s disease but also defines the role of rare variation in *NOD2* in the causation of Crohn’s disease.

## Materials and Methods

The purpose of this study was to identify highly penetrant, rare deleterious variants, to estimate the effect size of those variants in combination with common variants, and to estimate the impact of phase on effect size. Measures of effect size included relative risk estimates, percent of variance of disease explained, population attributable risk, and total heritability. Our approach included the identification of Crohn’s-affected children, their parents and a cohort of healthy controls. Prior to sequencing, we used genotyping arrays and principal components analysis to assure that cases and controls were of similar genetic ancestry. We then performed rare variant association tests and single marker association tests. We used logistic regression to estimate percent of variance explained, and meta-analyses to estimate population attributable risk.

### Subjects

The study included 205 Crohn’s disease cases with disease onset 18 years or younger. For 179 of these cases, trio sets were obtained by collecting samples from both parents. All subjects were phenotyped by one of three pediatric gastroenterologists with expertise in Crohn’s disease (SLK, LD, SLG). 60.1% of the subjects were male, and the median age of diagnosis was 11 years (see supplementary Figure S1). Controls were selected from healthy adult volunteers who had neither personal nor family history of autoimmune disease and no chronic diarrhea. The control group had a median age at enrollment of 23 years and 59.5% were male. The healthy adult controls have a greater time at risk than affected children, and therefore are less likely to carry disease-causing alleles. Additionally, phlebotomy in healthy children—especially under the age of 12 years–is difficult to justify ethically. Therefore, we deemed healthy adult controls as more appropriate controls. DNA was obtained from whole blood using standard procedures.

To identify population structure in cases and controls prior to sequencing, we generated 185,337 raw genotypes obtained for each study subject using the Immunochip genotyping array (Cortes and Brown 2011). To examine stratification in the data, the Immunochip data were merged with unrelated HapMap samples (60 CEU, 60 YRI, and 90 CHB/JPT). The overlap between data sets consisted of 17,119 SNPs. To eliminate platform-specific differences, A/T and G/C SNPs were removed. Additionally, SNPs with a minor allele frequency (MAF) less than 0.05 were removed. There were 13,549 SNPs remaining after all filtering. To select a genetically matched subset of samples for sequencing, we selected samples falling within three standard deviations of the centroid for the CEU HapMap cluster in a principal components analysis (PCA), as indicated by the red circle in Figure S2. The 200 control samples, who had no family nor personal history of autoimmunity, were randomly selected from 500 individuals for whom Immunochip data were available. Figure S2 displays the PCA with the final control set of 200 European Immunochip samples and the HapMap samples.

### Targeted capture and sequencing

We selected the coding regions of 97 genes for targeted sequencing based on their proximity to well-established GWAS associations. We also selected *STAT1* and *STAT4* because of our identification of *STAT1* as a cause of IPEX-like enteropathy and as positional candidates in a GWAS (Hu *et al.* 2014), and similarly, *STAT3* and *JAK2* because of their apparent involvement in Crohn’s disease pathophysiology(Willson *et al.* 2012). The full list of 101 genes is presented in Table S1. We used Agilent SureDesign to design probes for targeted enrichment using the HaloPlex Target Enrichment System. The targeted regions constituted 1,076 targets and 17,912 amplicons for a total of 497.3 kb of coding sequence.

Genomic DNA was diluted and digested using restriction enzymes. DNA fragments were hybridized to HaloPlex probes and sample indexes, captured using streptavidin beads, and ligated into circulated fragments per the manufacturer’s protocol. Target libraries were amplified and purified, and indexed samples were pooled for multiplexed sequencing. Sequencing libraries (25 pM) were chemically denatured and applied to an Illumina HiSeq v4 paired end flow cell using an Illumina cBot. Hybridized molecules were clonally amplified and annealed to sequencing primers with reagents from an Illumina HiSeq PE Cluster Kit v4-cBot (PE-401-4001). Following transfer of the flowcell to an Illumina HiSeq 2500 instrument (HCS v2.2.38 and RTA v1.18.61), a 125-cycle paired-end sequence run was performed using HiSeq SBS Kit v4 sequencing reagents (FC-401-4003).

### Bioinformatics and statistical analysis

#### Variant calling and quality control:

We processed targeted sequencing data from both case and control groups using GATK, which is a toolkit for SNV and inertion/deletion calling (McKenna *et al.* 2010). In brief, we used GATK HaplotypeCaller 3.2 to first obtain individual genotype calls, generating one gvcf file for each individual. We then used HaplotypeCaller to combine the gvcf files and jointly recall genotypes for all individuals. We filtered the joint-called variants to the targeted region and recalibrated according to the variants’ quality score (VQSR). We used 99.9 as the VQSR score cutoff for SNVs. We did not apply VQSR filtering to insertions and deletions (Indels) due to their limited number. We excluded all sites with missing genotype rates of 10% or greater in either the cases or controls. In total, 6,323 SNVs, 217 insertions, and 335 deletions were carried forward for subsequent analyses.

We tested for cryptic relationships using the relationship inference software KING(Manichaikul *et al.* 2010) and identified no first- through third-degree relationships among individuals in the study.

#### Association test and single-marker tests:

To evaluate evidence for rare variant association, we performed gene-based tests using the Variant Annotation, Analysis and Search Tool (VAAST), version 2.1(Hu *et al.* 2013). VAAST incorporates phylogenetic conservation and amino acid substitution information together with allele frequency differences between cases and controls to identify the set of variants in a given gene that are most likely to directly influence disease risk. A positive VAAST score indicates that the combined allele frequency and functional prediction evidence for the variant is sufficient to infer causality based on the Akaike Information Criterion. We constrained the population MAF of each variant in the likelihood model to 0.05 or lower (parameter –r 0.05). We restricted the VAAST analysis to the subset of 63 genes with five or more allele copies among cases and controls, which is the minimum number of allele copies needed to potentially achieve a p value of less than 0.05. For rare variants with MAF less than 1% in the controls, we conducted single-marker tests and estimated variant-specific odds ratios using non-Finnish European controls from the Exome Aggregation Consortium (ExAC) database(Lek *et al.* 2016).

#### Estimating effect size:

Using pre-specified covariates, we modeled the association between variants in *NOD2* and Crohn’s disease using logistic regression. We used two models to quantitatively investigate the variance of disease explained by common variants only, and common variants with additional rare variants and parental information. In the first model, we included the following covariates: heterozygosity for each of the L1007fs, R702W, and G908R alleles, and homozygosity for any one of these alleles. These data would be similar to the data obtained from a genotyping study without parental information. In the second model, we included the following covariates: heterozygosity for each of the L1007fs, R702W, and G908R alleles, homozygosity for any one of these alleles, any compound heterozygote (common and rare), and heterozygosity for any of the rare variants we deemed putatively disease-associated. This second model takes advantage of more information obtained by sequencing parents and rare variants obtained by sequencing. For both models, we included R702W because of its widely reported association with Crohn’s disease, although the variant was marginally significant in this study. Because of the quasi-separation noted for rare variants in cases, we used the method of Firth to estimate the maximum likelihood (Firth 1993). We approximated the variance explained from each model by calculating R^2^ using the method of Tjur (Tjur 2009).

For the meta-analysis of R702W, L1007fs and G908R, we included data from 89, 90 and 96 published articles(for R702W, G908 and L1007fs, respectively) with a combined total of 23,703 cases and 22,873 controls, which were published before December 31^st^, 2016(D’Addabbo *et al.* 2009; Schoultz *et al.* 2009; Yazdanyar *et al.* 2009; Csöngei *et al.* 2010; Dassopoulos *et al.* 2010; Gazouli *et al.* 2010; Glas *et al.* 2010; Hoffmann *et al.* 2010; Lacher *et al.* 2010; Sventoraityte *et al.* 2010; Yazdanyar *et al.* 2010; Naderi *et al.* 2011; Adeyanju *et al.* 2012; Azzam *et al.* 2012; Hama *et al.* 2012; Kanaan *et al.* 2012; Akyuz *et al.* 2013; Meddour *et al.* 2014; Boukercha *et al.* 2015; Salkic *et al.* 2015), and conducted the analysis in R using the ‘meta’ package. We calculated the odds ratios for the three variants in the meta-analysis using DerSimonian-Laird statistics as a random-effects model.

#### Population Attributed Risk and total heritability estimate:

We calculated the population attribute risk (PAR) for every potential risk variant using its odds ratio (OR) and allele frequency as the proportion of exposed subjects (Pe) from ExAC using the following formulas to account for the contribution from heterozygotes and homozygotes.(Witte *et al.* 2014). We converted the odds ratio to the relative risk (RR) using the Crohn’s disease prevalence (P0) of 34.7 cases per 100,000 children(Kappelman *et al.* 2013):

$$\;RR=\frac{OR}{\left(1-P0+(P0\times OR)\right)}$$

$$PAR=\frac{2Pe\left(1-Pe\right)\left(RR_{Bb}-1\right)+Pe^{2}(RR_{BB}-1)}{1+2Pe\left(1-Pe\right)\left(RR_{Bb}-1\right)+Pe^{2}(RR_{BB}-1)}$$

In this equation, RR_Bb_ and RR_BB_ are relative risk of heterozygotes and homozygotes for putative risk variant B. We calculated RR_BB_ under the assumption of a multiplicative model(Witte *et al.* 2014). We calculated the locus-wide PAR and its confidence interval using a multiplicative model rather than sum of individual PARs to obtain a conserved PAR(Natarajan *et al.* 2007; Goyal 2013). Finally, we also computed total heritability for putative variants using a web application, INDI-V (http://cnsgenomics.com/shiny/INDI-V/)(Witte *et al.* 2014).

#### *De novo* mutation analysis:

In addition to the variant-calling procedures outlined above, we also applied filtering criteria for *de novo* mutation calls(Kong *et al.* 2012). We also applied two additional *de novo* mutation filters, excluding all variants reported in ExAC with MAF greater than 0.5% in any population and all variants present in two or more individuals among our cases and controls. We conducted *de novo* mutation association analysis using VARPRISM, which integrates functional variant prioritization information to identify *de novo* mutations influencing disease risk (Hu *et al.* 2016) with the mutation rates in SNVs and indels of 1.2x10^−8^ and 4x10^−10^ per generation, respectively(Lynch 2010; Ségurel *et al.* 2014). Default settings were used for all other VARPRISM parameters.

### Data Availability

All sequences are available from the NCBI dbGap under BioProject accession phaXXXXX. Supplemental material available at Figshare: https://doi.org/10.25387/g3.6713156.

## Results

### Replication of established risk variants and identification of putative novel risk variants

Our gene-based rare variant case-control analysis identified three genes with nominally significant p-values: *NOD2*, *RTKN2*, and *MGAT3* (Table 1 and Supplementary Table S3.). Only *NOD2* was significant after Bonferroni correction (*P* = 1.53x10^−5^ x 63 = 0.00096). We identified 14 potential risk variants with positive VAAST scores in *NOD2*, including three well-established risk variants with MAFs of 1% or greater and 11 novel rare variants with MAFs less than 0.5% (Table 2). In addition to the 11 rare variants in *NOD2*, we also identified two variants with positive VAAST scores in *RTKN2*, and three in *MGAT3* (Table 2.).

**Table 1:** VAAST results for genes with nominal p-value less than 0.05

Gene	VAAST p-value	VAAST p-value after Bonferroni correction
*NOD2*	1.53x10^−5^	0.0009639
*RTKN2*	0.00165	0.10395
*MGAT3*	0.0147	0.9261

**Table 2:** Rare coding variants identified in *NOD2*, *RTKN2*, and *MGAT3*

	PolyPhen2 annotation	PolyPhen2 value	SIFT annotation	SIFT value	CADD	Allele frequencyin cases	Allele frequency in ExAC and controls	p-value
***NOD2* Variants**								
R702W	benign	0.008	deleterious	0	24.1	10.05%	3.502%	2.99E-09***
G908R	Probably damaging	1	deleterious	0	31	4.90%	1.407%	2.40E-06***
L1007fs	NA	NA	NA	NA	35	9.07%	2.020%	9.27E-14***
S47L	benign	0.98	deleterious	0.1	21.3	0.24%	0.12%	0.0886
E54K	probably damaging	0.995	deleterious	0.01	33	0.24%	0.003%	0.01825*
D291G	probably damaging	0.873	deleterious	0.01	23	0.25%	0.004%	0.02418*
R393C	probably damaging	0.996	deleterious	0	23.7	0.25%	0.004%	0.02396*
R420H	benign	0.082	tolerated	0.21	13.99	0.25%	0.001%	0.01209*
S431L	probably damaging	0.166	deleterious	0.03	21.4	0.24%	0.002%	0.401
P668fs	NA	NA	NA	NA	22.8	0.25%	0.002%	0.01220*
R791Q	benign	0.006	tolerated	0.22	0.074	0.49%	0.004%	0.2586
R830L	benign	0.006	tolerated	0.13	23.3	0.25%	0.001%	0.01212*
C842R	probably damaging	0.646	deleterious	0	23.4	0.25%	0.004%	0.02406*
C973R	benign	0.086	tolerated	0.36	16.96	0.25%	0.000%	0.00616^**a^
***RTKN2* Variants**								
A328fs	NA	NA	NA	NA	22.5	12.01%	16.399%	0.01561*
G573S	benign	0.001	tolerated	1	0.002	0.49%	0.106%	0.07243
***MGAT3* Variants**								
D499H	benign	0.409	tolerated	0.15	18.52	0.73%	0.670%	0.7559
R525W	benign	0.116	deleterious	0.01	22.4	0.25%	0.051%	0.194
R162Q	benign	0.004	tolerated	0.54	9.76	0.25%	0.024%	0.1319

* *P* < 0.05, **P < 0.01, ***P < 0.001; ^a^The p-value was estimated using the total NFE sample number at the nearby variant (chr16:50759433).

Given the rarity of these variants, we did not have sufficient power to evaluate each variant individually among our cases and controls. Therefore, we incorporated allele counts from the non-Finnish European sample (NFE) in the ExAC data together with our controls to conduct single marker association tests (Fisher’s Exact Test). The individual variant results should be interpreted with caution given the potential for systematic bias between cases and controls resulting from both population stratification and heterogeneous sequencing technologies.

**Figure 1:**
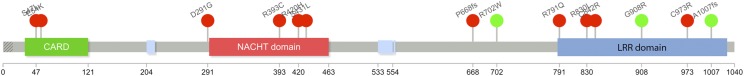
Location of risk variants in the *NOD2* protein. Green dots indicate the well-established Crohn disease variants; Red dots indicate the potential risk rare variants found in the VAAST result.

We identified 11 additional rare variants in *NOD2* (S47L, E54K, D291G, R393C, R420H, S431L, P668fs, R791Q, R830L, C842R and C973R) with positive VAAST scores suggesting that these rare variants occur in evolutionarily conserved regions of *NOD2* and result in more severe amino acid substitutions. These variants are novel potential Crohn’s disease risk variants, although S431L and R791Q were previously reported as likely benign variants in ClinVar with limited evidence of pathogenicity. Eight of the 11 variants were significant when we performed the single variant test using the allele counts in cases, and controls combined with ExAC data (*P* < 0.05): E54K, D291G, R393C, R420H, P668fs, R830L, C842R and C973R (Table 2). Although S431L was not significant, its odds ratio (OR:1.97; CI: 0.05-11.4) was comparable with a previous report (OR:1.45; CI:1.1-2.0) (Rivas *et al.* 2011). Six of the eight variants were identified as potentially damaging or deleterious by one or more functional prediction tools (Table 2)(Ng and Henikoff 2003; Adzhubei *et al.* 2010; Kircher *et al.* 2014). The scaled CADD score for each of these six variants was greater than 20, which represents the top 1% of predicted deleterious variants in the human genome. Nine of the 12 *NOD2* missense variants identified by VAAST were located within the boundaries of three *NOD2* domains: caspase recruitment domain (CARD), neuronal apoptosis inhibitor protein (NACHT) and leucine-rich-repeat (LRR) domain (Figure 1).

We observed two protein-coding *de novo* mutations among the 179 affected offspring, in *NOD2* and *C11orf30*. We tested for enrichment of *de novo* mutations using VARPRISM (Kong *et al.* 2012); both *NOD2* and *C11orf30* were nominally significant, with p-values of 0.0157 and 0.0175, respectively (Table 3).

**Table 3:** VARPRISM *de novo* mutation results

Transcript ID	Gene name	p-value	Number mutations	Genome Coordinate	Reference base	Alternative base
NM_022162	*NOD2*	0.015682657	1	chr16:50744999	C	T
NM_020193	*C11orf30*	0.017465753	1	chr11:76248871	C	G

### Replication of the common disease risk variant *ATG16L1 T300A*

Our single-marker tests replicated the association with the common susceptibility variant T300A (rs2241880) in *ATG16L1* (*P* = 0.0085). We also identified a rare coding variant in *ATG16L1* with an OR of 2.0, R258Q, although the variant was not statistically significant in the single marker test (Table 2). R258Q is a potential rare risk variant in Crohn’s disease because it was predicted to be a probably damaging variant in PolyPhen2 and had a high CADD score of 27. However, given the limited observations in our cases, further studies are required to understand the role of rare variants in *ATG16L*1 in Crohn’s disease.

### Effect size estimated for R702W, G908R and L1007fs in *NOD2*

Logistic regression models are shown in Table 4. In the first model, heterozygosity for G908R and L1007fs was associated with a 7.2- and 3.1-fold increased risk of Crohn’s disease, respectively. R702W heterozygosity was nominally associated with Crohn’s disease, and homozygosity for this allele was associated with a 6.5-fold increased risk of Crohn’s disease. The second model incorporated haplotype phase and rare variants. Heterozygosity for L1007fs, G908R and homozygosity for common variants remained statistically associated with Crohn’s disease. Compound heterozygosity was associated with a 6.2-fold increased risk of Crohn’s disease. The coefficient of discrimination (an approximation of R^2^ for rare events) for model one was 0.074 and for model two was 0.089, suggesting the ability to improve explained variance when incorporating rare variants and phase into explanatory models evaluating the association between variants in *NOD2* and Crohn’s disease.

**Table 4:** Logistic regression for three *NOD2* established variants

Variable	Case	Control	OR	Adjusted OR^1^	Adjusted p
**Model 1: R^2^= 0.074**					
L1007fs (heterozygous)	29 (14.2%)	10 (5%)	3.1 (1.5-6.6)	3.1 (1.4-6.5)	0.004**
R702W (heterozygous)	29 (14.2%)	19 (9.5%)	1.6 (0.8-2.9)	1.6 (0.9-3.0)	0.14
G908R (heterozygous)	16 (7.8%)	2 (1.0%)	8.4 (1.9-36.9)	7.2 (1.8-28.7)	0.006**
Any homozygote	12 (5.9%)	2 (1.0%)	6.2 (1.4-27.9)	6.5 (1.6-27.1)	0.01*
**Model 2: R^2^ = 0.089**					
L1007fs (heterozygous)	21 (10.2%)	8 (4%)	2.7 (1.2-6.3)	3.4 (1.5-8.0)	0.004**
G908R (heterozygous)	11 (5.4%)	4 (1%)	5.6 (1.2-25.7)	6.3 (1.5-26.4)	0.01*
R702W (heterozygous)	20 (9.8%)	17 (8.5%)	1.2 (0.6-2.3)	1.6 (0.8-3.2)	0.18
Common homozygote	12 (5.9%)	2 (1.0%)	6.2 (1.4-27.9)	6.8 (1.6-28.3)	0.008**
Compound heterozygote	12 (5.9%)	2 (1.0%)	6.2 (1.4-27.9)	6.8 (1.6-28.3)	0.008**
Rare heterozygote	5 (2.4%)	0 (0%)	11 (0.46-264)^a^	14.9 (0.6-360.5)	0.095

* *P* < 0.05, **P < 0.01, ***P < 0.001; ^a^ unadjusted OR for rare heterozygote and adjusted OR for all covariates are using the method of Firth (Firth 1993)

We further refined the effect size estimates of R702W, G908R and L1007fs by conducting a meta-analysis of our results together with prior studies (Yazdanyar *et al.* 2009)(see Supplemental Table S4-S6 for lists of studies). The per-allele odds ratios relative to healthy controls for R702W, G908R and L1007fs were 2.23, 2.58, and 3.83, respectively. The odds ratio for compound heterozygotes and homozygotes of the three variants was 7.31.

### Population attributable risk and total heritability for *NOD2*

We evaluated the population attributable risk (PAR) for the 14 coding variants in *NOD2* with MAF less than 5% (Table 2). We estimated that the three variants with MAF of 1% or greater (R702W, G908R and L1007fs) collectively confer 30.3% PAR (CI:17.0–45.2%) and 3.1% total heritability to Crohn’s disease. This PAR estimate was comparable with previous estimates from prior estimates ranging from 26.7 to 33.2% (Hugot *et al.* 2001; Abreu *et al.* 2002; Ahmad *et al.* 2002; Brant *et al.* 2007). For rare variants with MAF less than 0.5%, we restricted our analysis to the eight variants listed in Table 2 with significantly elevated allele frequencies in our cases relative to ExAC based on Fisher’s Exact Test. Together, these rare variants confer approximately 3.7% additional PAR (CI: 1.18–9.8%) and 0.25% total heritability explained. The total estimated PAR and total heritability for *NOD2* variants identified in our study were thus 32.9% and 3.4%, respectively.

## Discussion

We performed a sequence-based case-control study in 205 European children with Crohn’s disease incorporating 101 genes. Our study has three major findings. First, we again demonstrate that common and rare variation in *NOD2* represents the most important genetic risk factor for Crohn’s disease. Second, by sequencing trios, we identified compound heterozygosity and *de novo* mutations as potentially important sources for missing heritability. Third, we demonstrate the utility of applying gene burden tests such as VAAST to assess both common and rare variation in a complex genetic trait. Collectively, these data support the concept that the use of gene burden tests for complex traits and methods to incorporate phase such as sequencing parents and probands (as opposed to genotyping) can recover a portion of the “missing heritability” for Crohn’s disease.

The mechanistic relationship between gene variants in *NOD2* and Crohn’s disease pathogenesis is unclear, but could involve defective epithelial defenses, disruption of Paneth cell function, alterations in the gut microbial community, and/or defects in autophagy (reviewed in (Al Nabhani *et al.* 2017; Feerick and Mckernan 2017)). VAAST scored 11 variants in three key domains of *NOD2*: six in the LRR domain, two in the CARD domain, and three in the NACHT domain, including one *de novo* mutation. The NACHT domain is the same domain that harbors disease-causing mutations found in the majority of patients with Blau syndrome (Sfriso *et al.* 2012), a Mendelian disorder characterized by granulomatous arthritis, iritis and dermatitis. We speculate that patients with Crohn’s disease and mutations in the NACHT domain may represent an important, albeit uncommon, subgroup of patients potentially with phenotypic variability, variation in penetrance estimates for mutations in this region, variation in response to therapy, and/or intestinal disease distribution. Functional studies incorporating biological samples from subjects with NACHT domain variants, as well as a large cohort of such patients, are needed to test these hypotheses.

Our data suggest that at least some of the “missing heritability” for Crohn’s disease is explained by rare variants and haplotype phase. Unphased genotyping of the canonical variants in *NOD2* were assessed in our first logistic regression model, while we incorporated the phased data obtained from both parents and ascertainment of rare variants by sequencing in our second model, increasing the proportion of explained variance from 0.074 to 0.089. Similarly, PAR and total heritability estimates are increased from 30.3 to 32.9% and from 3.1 to 3.4%, respectively, when incorporating rare variants.

We acknowledge that our sample size is too small to fully characterize the contribution of extremely rare genetic variation in Crohn’s disease. However, we show the feasibility of using gene burden tests for Crohn’s disease sequencing studies by demonstrating that VAAST has the ability to uncover statistical association in *NOD2* among 63 genes and only 205 cases. We demonstrate the utility of using gene-based association tests rather than variant-based association tests as the field moves toward large-scale exome and whole genome approaches.
